# Triggering anger using a virtual reality social scene

**DOI:** 10.1038/s41598-026-36653-5

**Published:** 2026-02-21

**Authors:** Sinéad Lambe, André Miguel, Matthew Bousfield, Felicity Hudson, Memoona Ahmed, Ghizlane Slaoui, Phoebe Haynes, Aitor Rovira, Seena Fazel, Daniel Freeman

**Affiliations:** 1 Department of Experimental Psychology, University of Oxford, Oxford, UK; 2 Oxford Health NHS Foundation Trust, Oxford, UK; 3 Department of Psychiatry, University of Oxford, Oxford, UK

**Keywords:** Neuroscience, Psychology, Psychology

## Abstract

Virtual reality (VR) simulations may provide a safer way for people with high levels of anger to practise overcoming unhelpful reactions. The first step in such treatment development is to test whether anger can be caused by a simulation of a common triggering situation. The aim was to test whether an intimidating VR social environment raises anger in men and whether the effect is more pronounced in those with problematic anger. A mixed-design experimental study was conducted. Two hundred and sixty-five people were screened: 20 men were allocated to a high anger group based on the clinical cut-off of the Dimensions of Anger Reactions-5 (DAR-5) (> 12) and 22 men to a low anger group (DAR-5 score < 8). Anger was assessed pre- and post-exposure to an intimidating VR lift scenario featuring seven characters. Appraisals of the virtual characters were also assessed. Regression analyses were used to test the effects of anger group and appraisals on anger response whilst controlling for baseline anger. Across all participants, anger significantly increased from pre- to post-VR (*t*(41) = 3.63, *p* < .001, d = 0.56). There was a significant effect of group on the extent anger was triggered by the VR scenario (β = 0.277, *t*(df) = 2.443, *p* = .019, SE = 2.098) with the high anger group showing a greater anger response. Hostile appraisal of virtual characters (β = 0.416, *t*(df) = 3.285, *p* = .002, SE = 0.038) was associated with a greater anger response. The reactions to the virtual reality simulation mirrored what may be expected in the real world. A mildly stressful VR social situation raised anger in all but this was more pronounced in men who have difficulties with anger. VR has the potential to be used in the assessment and treatment of anger.

## Introduction

Immersive virtual reality (VR) comprises three-dimensional computer simulations of environments that can be navigated around and interacted with in a real way. It potentially provides a safe, controlled space for patients to learn at their own pace. Environments can be designed to match the situations patients find most challenging. Clinically-relevant environmental features such as types of people, facial expressions, and noises can be added to increase the potency of the experience. Tasks can be repeated and the level of difficulty gradated. Studies have shown that patients are more willing to enter challenging VR situations than the real situations and experiment with alternative ways of responding because they know the simulations are not real^1^. Crucially, the learning made in VR translates into the real world^2^. Randomised controlled trials have demonstrated the effectiveness of VR psychological therapy in treating anxiety-related difficulties such as phobias^3, 4^, posttraumatic stress disorder^5^, psychosis and agoraphobic avoidance^2^, and persecutory delusions^6, 7^. More recently, VR has been considered for the treatment of difficulties with anger and aggressive behaviour^8–10^. The success of VR for treating anger will depend on the effective activation of anger, and the associated cognitions, from simulations of common anger-inducing environments. We set out to show that a VR simulation of a mildly intimidating social situation can trigger anger and that the effects are greater in those who have problematic anger.

Anger is a normal human emotion, but is considered problematic when it is frequent, intense, and lasting, negatively affects social functioning, and is accompanied by a strong desire to harm the offending party^11^. Anger can be a common feature for a proportion of patients with post-traumatic stress disorder^12, 13^, borderline personality disorder^14^, depression^15^, panic^16^, psychosis^17^, eating disorders^18^ and substance misuse difficulties^19^ and is associated more severe symptoms and poorer treatment outcomes^20–25^. Problematic anger is associated with relationship problems across a range of settings^12^. In adolescence, poor anger regulation is associated with fewer friendships^26^. In adulthood, anger is associated with greater conflict in workplace and personal relationships. For example. in the workplace, anger is associated with taking revenge on co-workers^27^, and increased interpersonal and organisational deviance^28, 29^. Within personal relationships anger is also associated with greater use of antagonistic destructive behaviours^30^ as well as increase risk of interpersonal violence^31, 32^ and domestic violence^33^. As such, anger has been identified as a key treatment target in reducing violent behaviour.

Cognitive behaviour treatments are most commonly used in the treatment of problematic anger and aggressive behaviour. Strategies include increasing self-monitoring, identification and restructuring of cognitive biases (e.g. hostile appraisals), and development of arousal reduction strategies and interpersonal skills^34, 35^. Treatments for reducing anger have moderate effects in both non-clinical and psychiatric populations^36, 37^, whilst reports of the effectiveness of interventions for reducing aggressive behaviours have been inconsistent^37–39^. There is some evidence that interventions that include behaviour rehearsal have better outcomes^37^. One key advantage of virtual reality is that it can provide a low-risk environment to practise responding to the triggers of anger and aggression. This may be particularly useful for populations with restricted opportunities to practise new skills (e.g. forensic inpatients or prison populations) or those who, due to the risk of harm to others, cannot participate in behavioural interventions in real world settings.

Researchers have begun to develop VR therapies to treat difficulties with anger and aggressive behaviour, for example using VR to practise relaxation, address biases in social information processing (e.g. hostile appraisals), and to develop conflict resolution skills^8, 10, 40^. However, the success of such interventions will depend on the reliable activation of anger. There is little research demonstrating that VR can be used to activate anger and, if so, through what mechanisms. Most research to date has focused on the use of VR to elicit road rage. Studies indicate the validity of VR driving simulations in activating anger and dangerous driving behaviour^41, 42^. Two studies have illustrated the potential of VR to activate anger in social situations. Miyahira et al.^43^ randomised sixty non-clinical soldiers and military retirees to view anger provoking vignettes as a VR 360 video or on a flat screen. They demonstrated significant anger arousal in response to VR 360 video vignettes when compared to those viewed on a flat screen. Unlike immersive VR, 360 videos do not allow the user to actively participate in the scenario and instead the user is solely a spectator. Immersive VR may have greater applicability for skills development as it allows the user to actively participate in the scenario. One study did record anger levels during an immersive VR experience^9^. Sixty-male participants, divided into high and low aggression groups, completed scenarios where they had an argument with a virtual character. During these scenarios, participants were instructed to first express their anger (argument condition) and then to attempt to resolve the conflict (resolution condition). Users reported higher levels of anger in the argument condition compared to the resolution condition. There was no difference between the high and low aggression groups. To the best of our knowledge, no other studies have tested the use of immersive VR in activating anger.

The current study investigated the ability of an immersive VR simulation of a common social scenario to activate anger in individuals with anger difficulties. A virtual lift environment was designed to represent a mildly intimidating everyday social situation that could potentially trigger an anger response. Ambiguous or intimidating social situations likely elicit anger through social information processing biases including hostile appraisals of the behaviour of others^44, 45^. This study focused exclusively on men given the potential gender differences in triggers of anger^46–48^. For example, a man staring might be perceived as confrontational by a man and elicit feelings of anger; whilst it may be viewed as threatening for a woman and elicit feelings of fear. This study aimed to test three hypotheses. First, that the VR exposure would significantly increase self-reported anger. Second, that anger would be exacerbated to a greater degree for those with problematic anger compared to low anger controls. Finally, as hostile appraisals are highlighted as an important driver of anger^10, 35, 45^, we hypothesised that greater endorsement of the virtual characters as being hostile or judgemental would be associated with greater anger in response to the VR simulation.

## Method

### Participants

A mixed design was used to test the effect of exposure to a VR social environment on self-reported anger (within-subjects factor) and whether the effect of exposure differs for people with high versus low anger (between-subjects factor). Forty-two participants took part the study: twenty-two in the low anger group and twenty in the high anger. Participants were recruited through advertisements on social media and local radio stations. Those interested in taking part completed the study screening questionnaire which included the Dimensions of Anger – 5 (DAR-5)^49^. Inclusion criteria were being male and aged 18 or older. Participants were allocated to the high anger group if they scored above the clinical cut off (> 12) for problematic anger on the DAR-5. For the low anger group, participants who scored below this threshold were ranked by their DAR-5 scores, and those with the lowest scores were prioritized for invitation to participate to ensure maximal group differentiation.

### Procedure

Upon arrival, participants met individually with a research assistant and were informed that the study examined subjective experiences in virtual environments. To maintain experimental blinding, participants were not informed of the specific focus on anger responses. Participants first completed baseline questionnaires assessing anger. They also completed questions about anxiety, happiness, calmness, and sadness, to disguise the primary outcome measure. Additionally, participants completed a measure of paranoid ideation. Following questionnaire completion, the research assistant provided technical support for VR headset setup and ensured participant comfort with the virtual environment. Participants then completed the experimental virtual reality scenario. Immediately post-VR exposure, participants completed outcome measures assessing their subjective experience during the virtual scenario, including emotional responses and cognitive appraisals of virtual characters encountered within the environment. Following the assessment, participants were fully debriefed on the purpose of the study. All participants received twenty pounds as a compensation for their time in taking part in the study. Ethical approval was granted by the University of Oxford, Central University Research Ethics Committee (reference R81586/RE001). All participants provided informed consent to all study procedures. Partial disclosure of the specific study aims was used to minimise demand characteristics, and all procedures were conducted in accordance with the principles of the Declaration of Helsinki.

### Assessmentson

*The Dimensions of Anger Reactions* (DAR-5) is a five-item measure of anger assessing frequency, intensity, duration, aggression, and impact on social functioning^50^. Items are rated on five-point scales from none or almost none of the time (1) to all or almost all of the time (5). Higher scores indicate greater problematic anger. The DAR-5 has demonstrated good internal consistency, convergent, concurrent, and discriminant validity^49, 50^. A score of 12 or above on the DAR-5 indicates the presence of problematic anger that may benefit from clinical attention^49, 50^. The Cronbach’s alpha in the study sample (*N* = 42) was 0.89.

*Subjective Feelings of Anger Scale* (SFAS) was created for this study based on subjective units of distress (SUDS), which have been widely used in clinical psychology research and in previous studies validating the use of VR^6, 51, 52^. The scale has three items (rate how irritated you feel, rate how annoyed you feel, rate how angry you feel) assessing current feelings of anger. Each item is rated on an 11-point scale ranging from 0 (Not at all) to 10 (Extremely). Higher scores indicate greater feelings of anger. The Cronbach’s alpha in the study sample (*N* = 42) was 0.90.

*Appraisals of Virtual Characters Scale* (AVCS) was created for this study and includes 21 potential appraisal statements of virtual characters. Items were rated on a scale of 0 (did not believe) to 10 (completely believe). The measure has four subscales covering appraisals of characters as friendly (e.g. Everyone was pleasant), judgemental (e.g. They thought they were better than me), hostile (e.g. Someone was trying to intimidate me), and neutral (e.g. No-one had any particular feelings about me). Cronbach’s alpha in the study sample (*N* = 42) for each subscale was: friendly = 0.79, judgemental = 0.80, hostile = 0.96, and neutral = 0.77.

*Revised Green et al. Paranoid Thoughts Scale* (RGPTS) Part B^53^ is a 10-item self-report measure of persecutory ideation over the past month. Items are rated on a 5-point scale from 0 (not at all) to 4 (totally) with higher scores indicating greater paranoia. The Cronbach’s alpha in the study sample (*N* = 42) was 0.87.

### VR scenarios

**Fig. 1:**
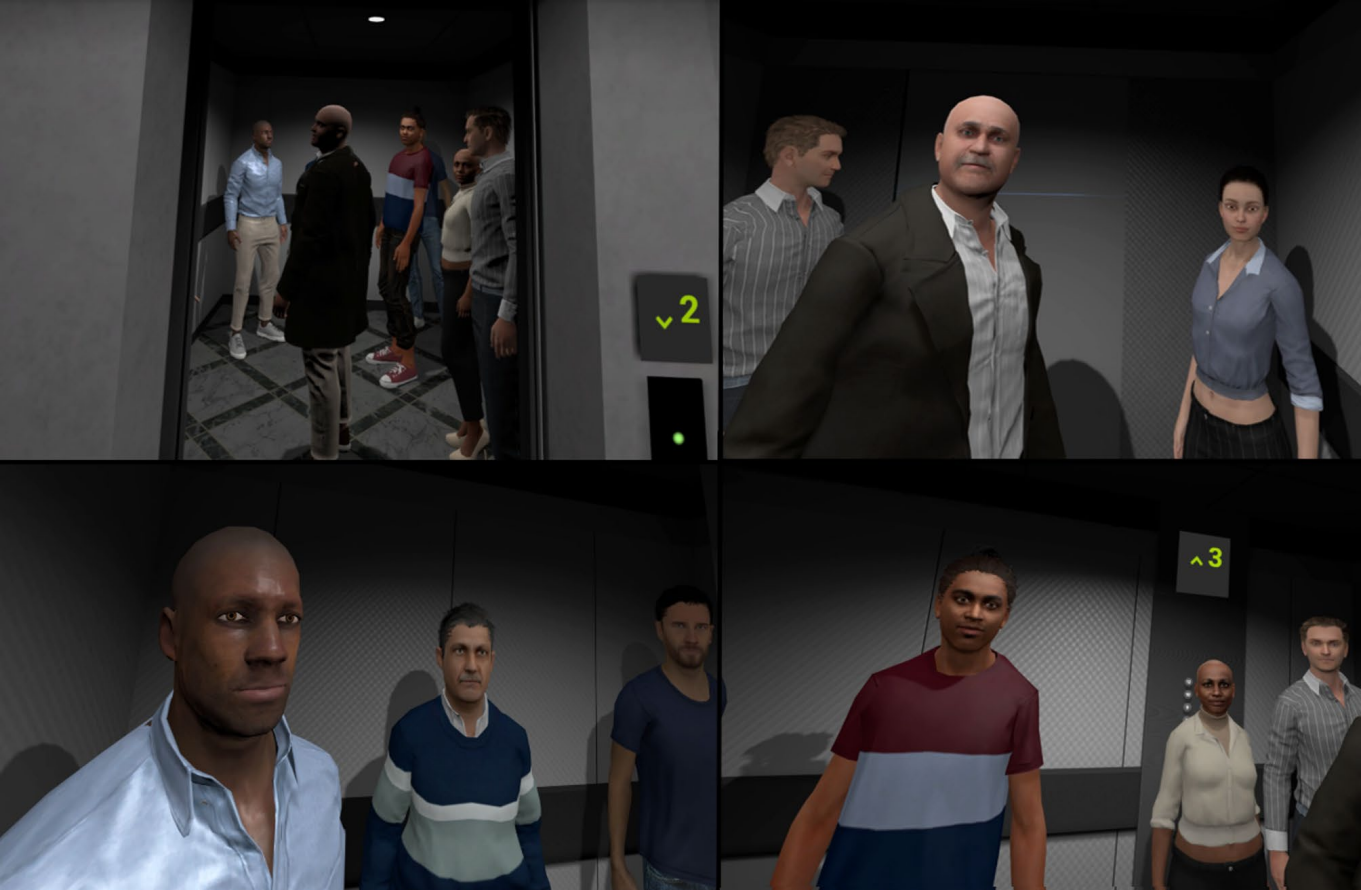
The lift scenario from the user perspective. Top left - View when the lift door opens. Top right – user view to their right in the lift. Bottom left – users view to their left. Bottom right- user view straight in front.

**Fig. 2:**
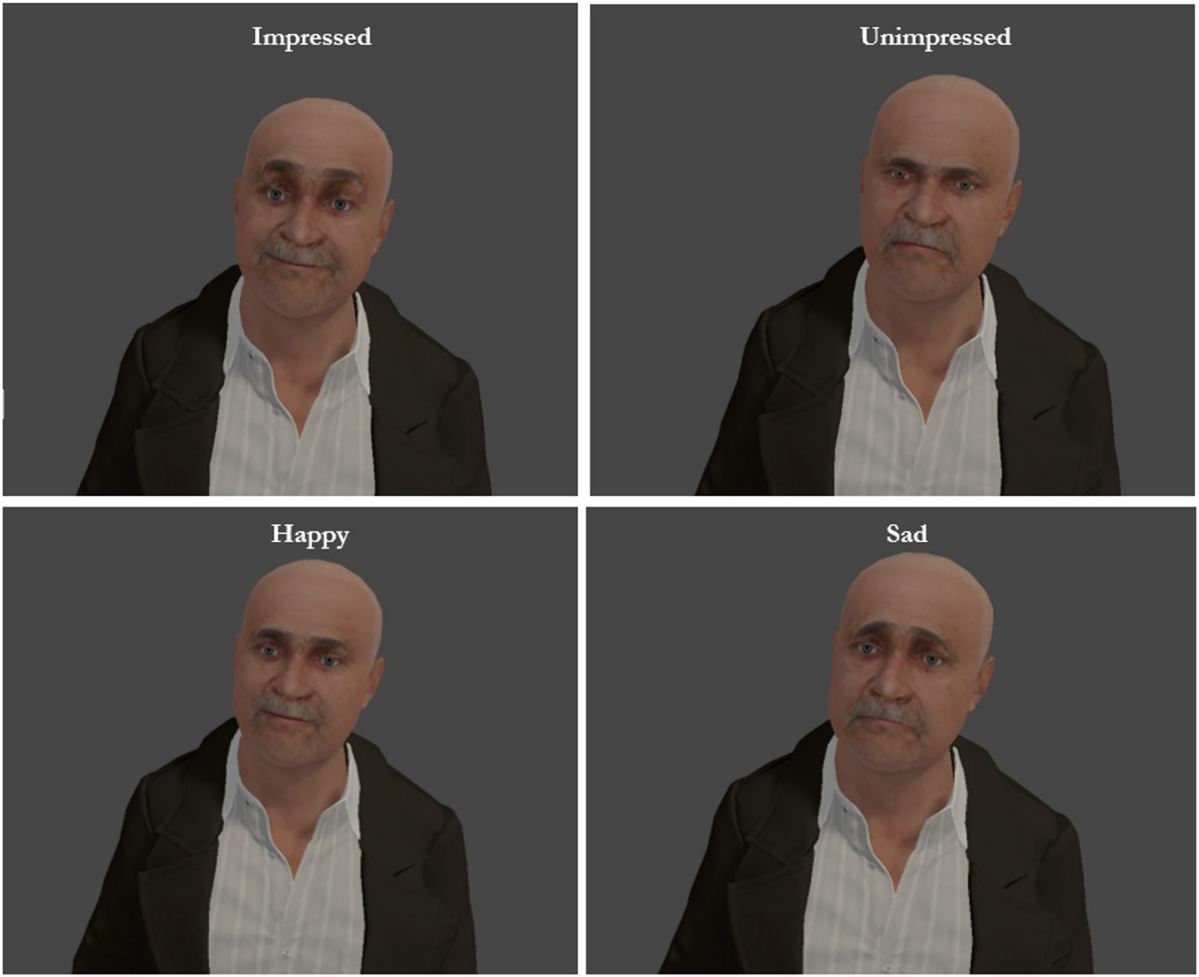
Examples of character facial expressions.

The VR experience was run with a Windows 10 computer (Nvidia GeForce GTX 1080Ti, 32 GB RAM) and rendered on a *Meta Quest 2* via Meta Air Link. Participants began in a foyer, waiting for a lift to arrive. The lift door opened automatically and the participant was teleported inside. The lift travelled to the top floor of a (very tall) building, which took approximately three minutes. The lift was dimly lit with seven characters inside (five male and two female). To create a mildly intimidating atmosphere three male characters were set to be taller than the user, eye contact was frequent, and the user was placed in a central position facing the characters (See Fig. 1). Facial expressions of the characters were set to be subtle and involved different combinations of happy, sad, impressed, and unimpressed (See Fig. 2).

### Statistical analysis

Analyses were conducted in SPSS^54^. There was no missing data. Before the main analysis a number of assumptions were assessed: linearity and homoscedasticity were examined via residual plots. Independence was evaluated using a Durbin-Watson test, with values near 2 indicating no autocorrelation. Normality was tested with Shapiro-Wilk (α = 0.05). Multicollinearity was assessed using VIF values (threshold < 5) and a correlation matrix. Influential observations were identified with Cook’s distance, using 4/(n-k) as the critical threshold.

To assess the effectiveness of VR in triggering an anger response, a repeated measures t-test was used to compare anger at baseline to anger after VR for the whole sample (hypothesis 1). The two groups were compared on all baseline and outcome variables using an independent sample t-test. A linear regression analysis examined the effect of group (low anger vs. high anger) on anger post VR when controlling for baseline anger (hypothesis 2). Finally, a linear regression analysis was used to examine the effect of appraisals on anger (hypothesis 3). Appraisals of virtual characters, group (low anger/high anger), and baseline anger were regressed upon anger during the VR scenario. Backwards elimination was used to remove non-significant appraisals from the regression model.

## Results

Two-hundred and sixty-five people were screened. Forty-two male participants completed the study (mean age in years = 43.0; SD = 15.6; range: 19–73). There were no incidents or adverse reactions to the VR scenario. Twenty-two participants were in the low anger group (mean DAR-5 = 5.36 SD = 0.72; range: 5–7) and twenty participants were in the high anger group (mean DAR-5 = 15.10 SD = 1.41; range: 12–17). The two groups differed significantly on baseline feelings of anger as assessed by the *Subjective Feelings of Anger Scale* (*t*(19.98) = 3.058, *p* = .006) with a mean difference of 3.63 (95% CI: 6.10 to 1.15) indicating that the high anger group reported greater baseline feelings of anger compared to the low anger group. See Table 1 for the participant characteristics and Table 2 for scores on all measures by group.

**Table 1:** Participant characteristics by group.

	Low Anger (*n* = 22)	High Anger (*n* = 20)
Age (years) Mean (SD)	44.64 (16.07)	41.25 (15.59)
Highest Education (n, %)		
GCSEs	1 (4.5)	1 (5.0)
AS Levels	0 (0.0)	1 (5.0)
A Levels	5 (22.7)	6 (30.0)
Bachelor’s degree	10 (45.5)	5 (25.0)
Postgraduate	6 (27.3)	7 (35.0)
Employment Status (n, %)		
Unemployed	2 (9.1)	4 (20.0)
Employed (full time)	7 (31.8)	13 (65.0)
Employed (part time)	3 (13.6)	1 (5.0)
Self-employed	4 (18.2)	2 (10.0)
Retired	4 (18.2)	0 (0.0)
Voluntary	1 (4.5)	0 (0.0)
Disabled/sick leave	1 (4.5)	0 (0.0)
Marital Status		
Single	7 (31.8)	7 (35.0)
Married/civil partner	13 (59.1)	8 (40.0)
Cohabiting	1 (4.5)	3 (15.0)
Separated	0 (0.0)	2 (10.0)
Prefer not to say	1 (4.5)	0 (0.0)
Ethnicity		
White British	12 (54.5)	10 (50.0)
Other White background	7 (31.8)	5 (25.0)
Mixed background	0 (0.0)	1 (5.0)
Asian - Indian	1 (4.5)	2 (10.0)
Asian - Chinese	0 (0.0)	1 (5.0)
Black - African	2 (9.1)	0 (0.0)
Other	0 (0.0)	1 (5.0)

Residuals showed a normal distribution (Shapiro-Wilk *p* = .156), observations were independent (Durbin-Watson *p* = .082), and no severely influential outliers were detected. There was moderate multicollinearity (highest VIF = 4.43) for hostile appraisals, however this was within the acceptable threshold.

### Hypothesis 1

A repeated measures t-test comparing anger at baseline (*N* = 42, M = 2.03, SD = 4.06) with anger post-VR (*N* = 42, M = 5.58, SD = 7.32) showed a significant increase following the VR t(41) = 3.63, *p* < .001, d = 0.56. This was a medium effect size.

### Hypothesis 2

**Fig. 3:**
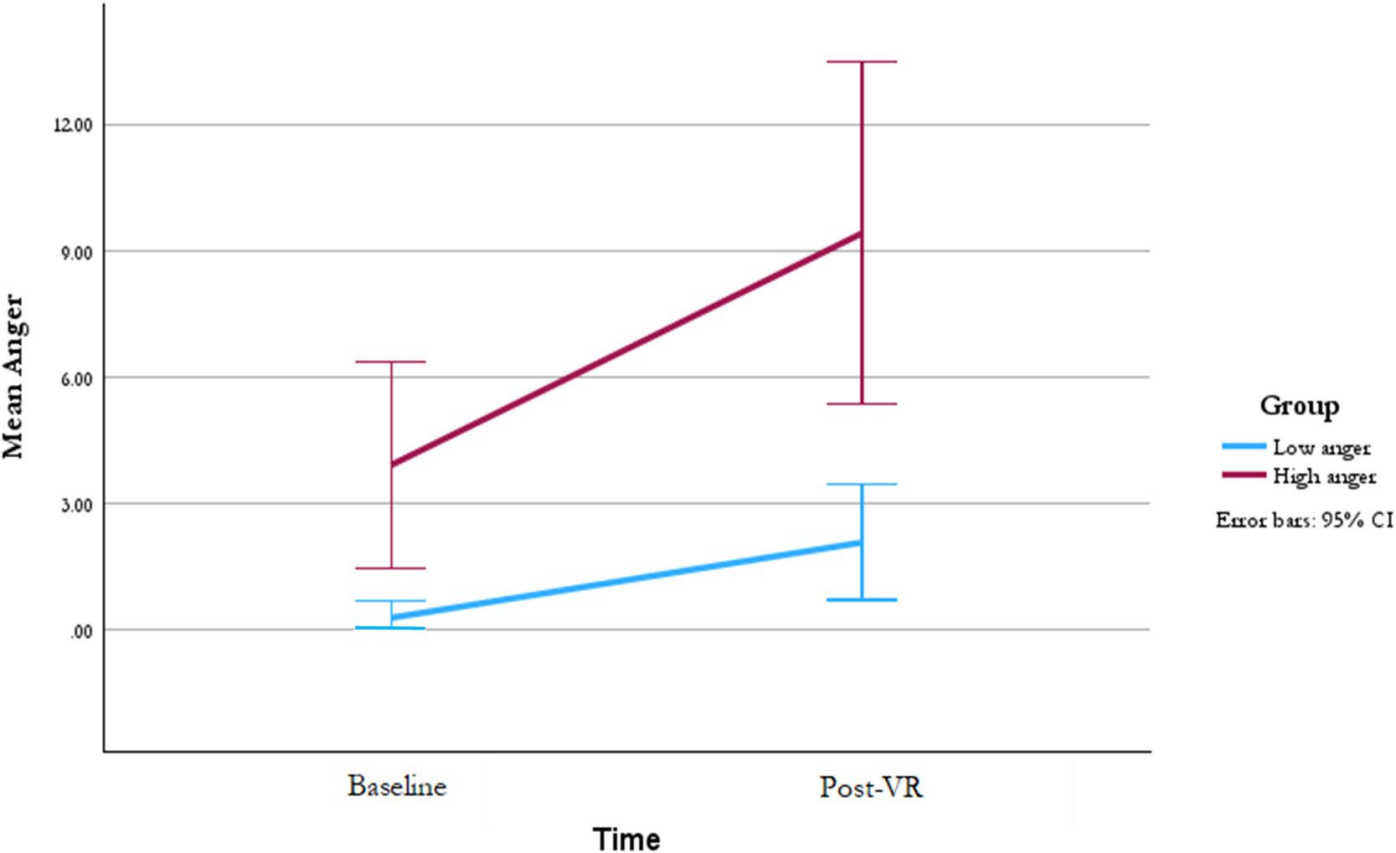
Mean anger scores by group across time points.

There was a significant difference on post-VR levels of anger in the low anger group (M(SD) = 2.09(3.09); range 0–13) compared to the high anger group (M(SD) = 9.43 (8.68); range 0–30) (see Table 2). A regression tested the effect of group (low anger vs. high anger) on anger post VR immersion whilst controlling for baseline anger. There was a significant effect of group on anger post-VR (β = 0.277, *t*(df) = 2.443, *p* = .019, SE = 2.098) whilst controlling for baseline anger, with the high anger group showing greater increases in anger compared to the low anger group (See Fig. 3; Table 2).

### Hypothesis 3

Using backwards elimination, a regression model reduced the predictors of anger in VR from six variables to three: group allocation (high anger vs. low anger), hostile appraisals of the virtual characters, and baseline anger. The model was statistically significant (F = 12.29, df = 3, 38, *p* < .001), explaining 49.2% of the variance in anger post VR. Endorsing hostile appraisals of the virtual characters was significantly associated with anger post-VR (β = 0.416, *t*(df) = 3.285, *p* = .002, SE = 0.038). There was a significant effect of group allocation on anger post-VR (β = 0.277, *t*(df) = 2.103, *p* = .042, SE = 1.907), whilst baseline anger showed a positive but non-significant relationship with anger post VR (β = 0.217, *t*(df) = 1.611, *p* = .116, SE = 0.243) but was retained in the model to control for initial levels of anger. Neutral, friendly, or judgemental appraisals were not significant and removed from the model. See Table 2.

**Table 2:** Outcome measures by group and group comparisons.

Variable	Low Anger M (SD)	High Anger M (SD)	t	*p*	Cohens D
Baseline					
Problematic anger (DAR-5)	5.36 (0.73)	15.10 (1.41)	27.71*	< 0.001	8.64
Anger (SFAS)	0.30 (0.88)	3.93 (5.24)	3.06*	0.006	0.97
Paranoia (RGPTS)	5.55 (7.37)	17.40 (12.03)	3.89	< 0.001	1.18
Post VR					
Anger (SFAS)	2.09 (3.09)	9.43 (8.68)	3.58*	0.002	1.13
Friendly appraisal (AVCS)	18.18 (10.35)	13.67 (9.46)	-1.47	0.150	-0.45
Neutral appraisal (AVCS)	15.39 (9.72)	13.68 (11.40)	-0.52	0.603	-0.16
Judgmental appraisal (AVCS)	14.78 (9.72)	17.13 (10.65)	0.75	0.461	0.23
Hostile appraisal (AVCS)	27.19 (22.47)	42.54 (24.60)	2.11	0.041	0.65

Note: * indicates Welch’s t-test used due to unequal variances (Low Anger *n* = 22, High Anger *n* = 20). Effect sizes: Small (≈ 0.2), Medium (≈ 0.5), Large (≈ 0.8).

## Discussion

This study examined the effectiveness of a virtual reality (VR) environment in eliciting anger and tested whether this effect was more pronounced among individuals who experience problems with anger. The VR environment was designed to be intimidating through subtle non-verbal social cues such as prolonged eye contact, facial expressions, and close proximity in an enclosed space. The findings indicated that this VR environment could significantly increase anger, though the intensity of the anger response was moderate. As hypothesised, individuals high in problematic anger showed a stronger effect, even after controlling for baseline anger levels. Hostile appraisals of virtual characters were clearly generated in the high anger group and were associated with heightened feelings of anger during the VR experience. In addition, there was a large difference in paranoia between the high and low anger groups. It suggests that the general belief that others intend to do you harm will inform in the moment hostile appraisals of ambiguous social cues, a process that may be important in anger.

These findings are consistent with research showing that individuals with high levels of problematic anger often exhibit heightened emotional reactivity^55, 56^. The association between hostile appraisals, anger and aggression is an established finding in the literature^45^ but this is, to the best of our knowledge, the first study to replicate this finding using VR. The effect of group allocation remained significant even after controlling for hostile appraisals, indicating that the greater anger responses in the high anger group was not fully explained by differences in threat perception. This may suggest the involvement of additional mechanisms—such as poor emotion regulation, increased autonomic reactivity, or maladaptive responses to provocation^31, 55, 57^.

The successful activation of anger and hostile appraisals in VR provides support for the potential of VR in both the assessment and treatment of anger difficulties. Developing such VR tools will require a strong theoretical understanding of anger. The high anger group showed significant variability in anger intensity during VR. This highlights that the triggers of anger are likely heterogeneous and may encompass a range of factors including hostile attribution bias, goal frustration, in-group outgroup dynamics, sensory overload (e.g. crowds, noise), and overt mistreatment by others (e.g. unjust, disrespectful, or threatening behaviour). Knowledge of the key triggers of anger will be essential for future VR treatment development. Our findings validate the use of VR to test theoretical models of anger and examine techniques for the manipulation of anger. This approach could form the foundation for treatment development using VR.

The current study has a number of limitations. It included only male participants. This was a deliberate methodological choice based on well-established gender differences in threat perception and emotional responses^46, 47^. Thus, the study needs to be replicated with female participants to examine whether similar mechanisms and responses are observed across genders. VR is generally considered a safer way to elicit difficult emotions^10, 58^. In line with this there were no incidents reported during the current study. However, the safety of using such VR applications with high-risk populations (e.g. forensic or prison populations) needs to be assessed further. The study used self-report measures of anger which may be influenced by social desirability. In addition, the assessments were developed for the purpose of this study and thus have not been validated. Future studies would benefit from including physiological measures, which would provide objective assessments of arousal.

This study demonstrates the capacity of VR to elicit an anger response, yet more research is needed to identify the key conditions for provoking anger and its underlying mechanisms. VR offers a novel opportunity for advancing our theoretical understanding of anger and the development of innovative targeted interventions.

## Data Availability

Data are available upon reasonable request. Deidentified participant data will be available in anonymised form from the corresponding author (SL) on reasonable request (including a study outline), subject to university approval and contracting.
